# Comments on: Interpretation of genome-wide infinium methylation data from ligated DNA in formalin-fixed paraffin-embedded paired tumor and normal tissue

**DOI:** 10.1186/1756-0500-5-631

**Published:** 2012-11-13

**Authors:** Christina Thirlwell, Andrew Feber, Matthias Lechner, Andrew E Teschendorff, Stephan Beck

**Affiliations:** 1 Medical Genomics Laboratory, UCL Cancer Institute, 72, Huntley Street, London WC1E 6BT, UK

## Abstract

BMC Research Notes recently published a research article regarding the use of ligated DNA extracted from formalin-fixed paraffin embedded (FFPE) tissue on the Illumina Infinium methylation platform - “Interpretation of genome-wide infinium methylation data from ligated DNA in formalin-fixed, paraffin-embedded paired tumor and normal tissue” Jasmine *et al.* BMC Research Notes 2012, **5**:117. This article repeatedly refers to our previous work and concludes that methylation data obtained from ligated FFPE extracted DNA should be used with great caution. In this Discussion we review the data analysis performed in Jasmine *et al’s* paper and suggest limitations which subsequently lead the authors to draw what we believe are incorrect conclusions. Moreover, we continue to analyse genome-wide methylation data from DNA extracted from FFPE tissue successfully on both the HumMeth27 and 450 K arrays.

## Discussion

We are writing in response to the recently published research article “Interpretation of genome-wide infinium methylation data from ligated DNA in formalin-fixed, paraffin-embedded paired tumor and normal tissue” Jasmine *et al.* BMC Research Notes 2012, **5**:117 [1].

Throughout the article reference is made to our previously published paper describing a novel method for the analysis of formalin-fixed, paraffin embedded (FFPE) extracted DNA on the Illumina HumMeth27 DNA methylation array [2]. We feel that there are at least four major limitations concerning the statistical analysis implemented in Jasmine *et al’s* paper, which lead to the conclusions drawn to be overly pessimistic.

### Batch effects

One issue which Jasmine *et al.* acknowledge in their Discussion is that of a potential batch effect confounding their analysis. This batch phenomenon has been described in the analysis of HumMeth27 data by Teschendorff *et al.*[3] and Leek JT *et al.*[4]. As pointed out in Leek JT *et al.*, it is not uncommon that over 50 to 80% of measured probes may be subject to confounding by batch effects. In Jasmine’s paper, all of the fresh frozen (FF) samples were analysed on a separate date/batch to the FFPE samples, hence it is not entirely surprising that the largest variation is associated with batch, and that consequently the correlations between the matched FF and FFPE samples is lower than expected. Moreover, since batch and FF/FFPE status are completely confounded (in our view a fundamental limitation of their study), it is simply wrong to draw the conclusion that the differences seen are entirely driven by FF/FFPE status.

### Differentially methylated loci (DML) analysis

Jasmine *et al.* imply that among the top 50 DMLs from the FFs and FFPEs there are “only” 7 in common. This is without determining whether 7 is significant or not. In fact, a simple binomial test shows that 7 is greater than would be expected by random chance: under the null, the expected number of overlaps would be approximately 50*(50/27000) = ~0.1+/−0.6, that is, on average we would expect no CpGs to overlap among the corresponding top 50 DMLs. The P-value from the Binomial test for an overlap of 7 is P < 1 × 10^-10^, i.e this is highly significant. In our experience, that among the top 50, there are 7 loci that overlap (i.e a 14% (7/50) overlap), reflects a relatively strong agreement exactly in line with the fact that the FF and FFPE samples were derived from the same patients and given that they were only comparing 12 samples in each phenotype. Therefore, that the overlap is not stronger (i.e over 50%) is simply down to a lack of power. This should not be surprising since as shown in Ein-Dor *et al.*[5], the ranking of features in “omic” studies becomes stable (i.e over 50% overlap) only when relatively many samples are analysed (>100 samples).

### Data analysis methodology for calling DML’s

The overlap of DMLs between the FF and FFPE sets might be even larger if the authors had used a Bayesian state of the art algorithm for detecting DMLs. In fact, Jasmine *et al.* used the Illumina in-house model for calling and ranking DMLs, which estimates variance from an in-house (and therefore independent) experiment. Hence, the variance/noise estimates used by the authors do not reflect the variance/noise estimates of their own data, a key point which will definitely have affected the ranking of the DMLs. Given that the authors were only comparing 12 versus 12 samples, it would be more accurate to have called and ranked DMLs according to a Bayesian framework with locus-specific variances estimated from the data itself, as implemented for instance in the limma R package [6].

### Concordance evaluation

Generating scatterplots of –log10 (p-values) between the FF and FFPE sets is inadequate for evaluating concordance. P-values are highly unstable and don’t reflect the directionality of methylation changes. Jasmine *et al.* should have generated a scatterplot of the statistics of differential methylation (e.g the regularized t-statistic for DMLs (cancer minus normal)) for FFs against the corresponding statistics obtained using FFPEs, all derived using an empirical Bayes framework [5]. It is likely that this would have revealed a strong agreement in terms of the broad statistical significance and directionality (i.e. hyper/hypo methylation) of DMLs.

We continue to successfully analyse FFPE extracted DNA on the Illumina HumMeth platform and have refined the original published method [2] which has improved the performance of these samples (manuscript in press). We have also tested the Illumina FFPE DNA sample QC kit which has been developed specifically for this assay and tested in-house methods alongside this.

In conclusion, we suggest that should Jasmine *et al.* have analysed their data as set out above and designed their experiment to eliminate/minimise batch effects, their findings would demonstrate the effective use of DNA extracted from FFPE tissue on the Illumina HumMeth platform.

## Response

A signed response to comments on: Interpretation of genome-wide Infinium methylation data from ligated DNA in formalin-fixed paraffin-embedded paired tumor and normal tissue.

Farzana Jasmine^1^, Habibul Ahsan^1^, Mohammed Kamal^2^, and Muhammad G Kibriya^1*^

E-mails: farzana@uchicago.edu; habib@uchicago.edu; kamalzsr@yahoo.com and kibriya@uchicago.edu

Address:

^1^ Department of Health Studies, University of Chicago, Chicago, IL 60637, USA

^2^ Department of Pathology, Bangabandhu Sheikh Mujib Medical University (BSMMU), Dhaka 1000, Bangladesh.

^*^Corresponding Author: kibriya@uchicago.edu

In our recently published paper [1], we have shown genome-wide methylation data derived from modified Infinium protocol using ligated FFPE DNA as was originally shown by Thirlwell *et al.*[2]. It may be noted that Thirlwell *et al.*[2] did not compare tumor and normal tissue to identify tumor specific DML from FF and FFPE tissue. Our data suggested that the tumor specific DML detected in FFPE samples are not quite the same as the tumor-specific DML detected from FF tissue (gold standard) from exactly the same patients. Based on our findings we concluded that Infinium methylation data from FFPE should be interpreted cautiously. We thank Thirlwell *et al.* for their interest in our paper and commenting on the following issues:

**Batch effect:** We acknowledge that in the presence of batch effect there is potential for finding some false tissue specific DML (FF vs. FFPE). However, the fact that all the FF samples from tumor and normal were analyzed in a single batch and paired samples were put on same chip (same for FFPE samples) there should not be any batch effect while detecting the tumor-specific DML. Unfortunately, we found largely different sets of tumor specific DML while using FF and FFPE samples. It may be mentioned that in our experience Infinium methylation assay is very reproducible and inter-batch replicate (done in two different labs at different time) gave us very strong correlation (r = 0.96 to 0.98) [7].

**DML analysis:** we have shown the actual numbers and/or percentage of the overlapping tumor specific DML derived from FF and FFPE samples. We agree that statistically speaking 7 loci out of top 50 DML (14% overlap) being shared among the two lists is unlikely to be due to chance, however we leave the importance of this overlapping proportion to the readers and researchers who would like to clinically/biologically interpret the results. We also looked at the performance of modified Infinium protocol on FFPE samples for the usual suspects (frequently found DML in tumor) - see Additional file 6: Table S2 in the original manuscript) [1]. Results were not satisfactory for FFPE. Regarding sample size and power of the study, analysis of our own methylation data suggested that we had >90% power to detect a 1.25 fold change between tumor and normal groups (in other words a difference of methylation beta value of 0.1 and 0.125 or between 0.5 and 0.625) both in FF and FFPE samples.

**Methodology for calling DML:** It may be noted that Thirlwell *et al.* have somehow overlooked the fact that we used multivariate ANOVA to detect the DML. We also showed results from paired *t*-test and Bootstrapping (see Additional file 3: Figure S3 of original paper) [1].Therefore their comments regarding the variance/noise estimates does not apply to our data analysis. We acknowledge that we did not use Bayesian framework, but all three statistical tests that were applied to identify DML in our paper are pretty standard and statistically appropriate for the type of research question and there was substantial overlap between them (see Additional file 3: Figure S3 of original paper [1]) indicating that we picked up the true DML [1]. Previously we also have seen very good overlap between results from Illumina’s DiffScore and results based on the multivariate ANOVA [8] and therefore could not completely agree with their criticism of DiffScore analysis. Vast majority of true DML should survive any valid statistical tests applied to the data [8].

**Figure 1:**
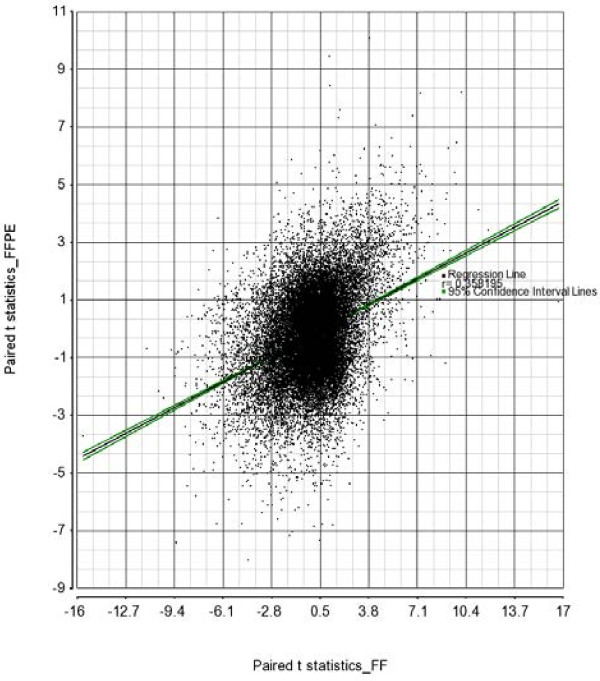
**Scatterplot of t-statistics from paired *t*-test between tumor and corresponding normal tissue from FFPE samples (y-axis) and FF samples (x-axis) from the same patients.**

**Concordance:** We agree with Thirlwell *et al.* that p-value is often unstable. In fact we have tested the research question from different angles. The scatterplot from –log p-value was shown in additional material, but in the main body we also showed the loci specific correlation between FF and FFPE (see Figure six in the original manuscript [1]) for all the 27 K loci [1]. In our data, we could not see meaningful strong correlation. As per the suggestion by Thirlwell *et al.*, we have now added the scatterplot of t-statistics from paired *t*-test between tumor and normal in FFPE (y-axis) over FF (x-axis) in Figure 1, which also does not show strong concordance between FF and FFPE results. In the original paper we went further to examine the effect of ligation on FFPE DNA by testing the performance on SNP genotyping platform. Although call rate was not very low, the performance was significantly poor than FF samples [1]. Performance of FFPE samples on DASL assay was also not optimal [9]. Although somewhat pessimistic, our words of caution for interpretation of genome-wide data from Illumina Infinium platform are based on our microarray data.

In fact we, like other cancer researchers, would be very pleased if one could get identical biological information regarding tumor specific DML or gene from widely available FFPE samples as one would expect from FF samples from same individuals. Unfortunately so far no published paper has clearly demonstrated that. It would have been better if Thirlwell *et al.* presented some real data showing tumor specific DML from FF and FFPE samples in their signed response.

Lastly, our entire data is submitted to GEO and will be released in due course. So, researchers are welcome to apply any statistical tests that they feel appropriate and make their own conclusions. Recently Illumina has further modified the assay by adding another step to restore FFPE DNA before ligation step for methylation assay. We are yet to evaluate that kit to investigate if FFPE samples can provide similar information as FF samples.

## Competing interests

The authors declare that they have no competing interests.

## Authors’ contributions

CT and AET drafted and edited the article. AF, ML and SB provided critical revision. All authors have approved the article prior to submission.
